# Bladder Cancer: Current Challenges and Future Directions

**DOI:** 10.3390/medicina57080749

**Published:** 2021-07-24

**Authors:** Jakub Dobruch, Maciej Oszczudłowski

**Affiliations:** Centre of Postgraduate Medical Education, Department of Urology, 00-416 Warsaw, Poland; moszczudlowski@cmkp.edu.pl

**Keywords:** urinary bladder cancer, surgical treatment, BCG failure, perioperative chemotherapy

## Abstract

Bladder cancer (BCa) is the most common malignancy of the urinary tract and one of the most prevalent cancers worldwide. While the clinical approach to BCa has remained largely unchanged for many years, recent discoveries have paved the way to a new era of diagnosis and management of the disease. BCa-specific mortality started to decrease in the regions with a wide range of activities leading to greater social awareness of the risk factors and the decline in carcinogenic exposure. The urologic community refines the role of transurethral surgery towards more rigorous and high-quality techniques. New agents have been approved for patients with BCG failure who faced radical cystectomy so far. Although radical removal of the bladder is the gold standard for muscle invasive cancer management, the extent and clinical value of lymphadenectomy is currently heavily challenged in randomized trials. Furthermore, alternatives to perioperative chemotherapy have arisen to increase the likelihood of complete treatment delivery and successful oncological outcomes. Finally, improvements in molecular biology and our understanding of tumorigenesis open the era of personalized medicine in bladder cancer. In the present review, the status and future directions in bladder cancer epidemiology, diagnosis and management are thoroughly discussed.

## 1. Introduction

Bladder cancer (BCa) remains the most common malignancy of the urinary tract. In 2018, BCa was diagnosed in 549,393 patients and 199,922 succumbed to the disease worldwide [1]. Although age-standardized incidence (ASIR) shows great diversity among different geographical regions, it is projected to continue the rise within the next decade [2]. A number of BCa risk factors were identified. Apart from geography and age, the risk varies between genders and is heavily influenced by exposure to a number of carcinogens, with cigarette smoking being the most prevalent one [1]. Age-standardized mortality rates (ASMR) started to decline in well-developed countries, while it tends to increase in low-income parts of the globe [3].

The most significant symptom of BCa is either microscopic or gross hematuria. If the bladder tumor is identified, in 75% of cases, urothelial bladder cancer (UBC) confined to the mucosa (NMIBCa—non-muscle invasive disease) is diagnosed [4]. In the remaining 25–30% of patients, BCa has already invaded deeper layers of the bladder wall (MIBCa—muscle-invasive disease) or formed metastases. Transurethral resection of the bladder tumor (TURBT) is the mainstay therapy of those with NMIBCa, whereas radical removal of the bladder (RC—radical cystectomy) is implemented in those with MIBCa. To prevent recurrence and progression, TURBT is supplemented with intravesical instillations in select patients. Despite a number of surgical and anesthesiological improvements and wide adoption of perioperative chemotherapy, the long-term survival rates of patients with UBC have remained unchanged for decades. At the same time, our understanding of the disease biology by advanced molecular research has increased substantially. It is believed that therapy will be individualized and together with several refinements of surgical techniques and novel systemic as well intravesical treatment modalities will lead to better oncological outcomes of our patients. In this review, selected challenges and imminent perspectives with respect to bladder cancer epidemiology and management are presented and discussed.

## 2. Evidence Acquisition

A nonsystematic review of the PubMed database to identify articles focused on current challenges and future directions published in recent years in the English language was conducted. The following phrases were used during the search: urothelial cancer or bladder cancer, combined with several groups of keywords relevant to the discussed sections. These included: biology, etiology, epidemiology, management, and outcomes. Clinical series, review articles and editorials were identified, and all abstracts were reviewed but only the most significant papers were completely analyzed and used as references. 

## 3. Evidence Synthesis

### 3.1. Epidemiology

According to WHO reports, the incidence of BCa varies significantly among different geographical regions, such that age-standardized rates are almost three times greater in more developed areas than in less prosperous countries (9.5 vs. 3.3). The variety is among others suggested to result from disparities in tobacco smoking, obesity, alcohol consumption and overuse of red meat. Although the BCa risk is multifactorial, cigarette smoking remains the most prevalent risk factor. It has been estimated to be responsible for half of all BCa cases [5], with the magnitude of harms corresponding to smoking intensity and duration [6]. According to recent analysis, strong correlations between tobacco use and BCa incidence as well as mortality exist, though these are observed in males to a greater extent than females in both measures [3]. The WHO has issued a warning concerning the high prevalence of cigarette smoking and beforementioned risk factors across the European countries and at the same time provided number of pivotal interventions that policy-makers should implement to reduce the exposure [7]. Among many, the most important include the improvement of population awareness by comprehensive education campaigns, smoking ban in public places, increasing the price of cigarettes and alcohol, enhancing supermarket visibility of fruits and vegetables as well as their consumption, promoting physical activity by different measures such as stairs instead of lifts in attractive buildings and stimulating cycling as the most convenient means of transportation, restricting food advertising in media directed at children, banning industrially produced trans fatty acids and increasing duty on petrol [7]. Although on a global scale, rates of tobacco use are declining, the target of a 30% reduction by 2025 set by the WHO Global Action Plan for the Prevention and Control of Noncommunicable Diseases 2013–2020 is likely not to be achieved in the majority of countries. In the meantime, decreasing the prevalence of cigarette smoking in European countries is finally followed by a slow reduction in BCa-specific mortality [8]. The decline, however, does not result solely from the decrease in smoking pervasiveness but also from easier and quicker access to high-quality medical care, leading to early detection of potentially deadly disease. Analysis of the incidence of BCa throughout recent years in high-income countries has shown increasing tendencies with a corresponding decline in BCa-specific mortality [3]. 

There is no widely accepted BCa screening program. This is probably due to the low incidence of aggressive disease and lack of an optimal screening tool. Screening has been suggested, however, to be efficacious in highly selected populations. Those known to have aristolochic acid nephropathy were subjected to biannual cystoscopies for 10 years, and in half of them, BCa was diagnosed. After a median of 94 months follow-up, nobody died of the disease [9]. Therefore, the identification of vulnerable subpopulations that should be embraced by the appropriate screening program is crucial. 

Occupational exposures have long been associated with bladder cancer risk. While professions at the greatest risk include industrial areas processing paint, rubber, petroleum products and dye workers, the greatest risk of BCa specific mortality is observed in electrical and chemical process workers [10,11]. Of all BCa cases, no more than 8% are found to be attributable to such exposures [12]. However, some workers may be notably more prone to dismal effects of these subjections. Recent genetic study has revealed two carcinogen-detoxification genes NAT2 and GSTM1 that when abnormal lead to longer exposure to carcinogens [13]. As such, translational studies combining genetic profiles with environmental and occupational carcinogen exposures to reveal the targets for prophylactic interventions are warranted. 

### 3.2. Non-Muscle Invasive Bladder Cancer

Transurethral resection of the bladder tumor (TURBT) is the mainstay therapy for non-muscle invasive disease. This surgery is performed with resectoscope that is used to cut the lesion into pieces subsequently directed for pathology. TURBT has been heavily criticized due to its piecemeal technology that may lead to tumor cells implantation within healthy bladder mucosa. Single post-TURBT chemotherapy instillations were found to decrease the recurrence risk in NMIBCa cases, explicitly seen in primary, single lesions [14]. Therefore, en bloc bladder tumor removal (EBRT) has been introduced and is gaining recognition worldwide. It is supposed to limit cells spillage, decrease the risk of bladder perforation and provide enough tissue material to reliably evaluate the margins status [15]. Surprisingly, in comparison to standard resection, EBRT has not been proved to significantly reduce the recurrence risk and is limited in multifocal, flat lesions, especially those located at the bladder dome [16]. Furthermore, transurethral removal of tumors larger than 3 cm after EBRT remains a challenge. 

NMIBCa has been observed by many investigators to recur and progress. Five-year probability of recurrence and progression mounts to 78% and 45%, respectively [17]. Several efforts were undertaken to improve the prognosis. Technological innovations leading to enhanced visualization of abnormal bladder mucosa lesions including narrow-band and photodynamic imaging were introduced and were shown to increase the diagnosis of Cis and decrease the risk of early BCa recurrence. However, the quality of TUR itself has been recognized by some authors to be one of the most important factors in NMIBCa diagnosis and management. In 2008, to address the need for cancer care improvement, the Scottish government started Scotland’s Quality Performance Indicator (QPI) programme. According to its recent report, recurrence risk at first follow-up cystoscopy in low-risk NMIBCa and the probability of residual disease in high-risk NMIBCa have dropped by 12% and 4%, respectively, through the years 2014–2017 [18]. Despite these improvements, patients with NMIBCa face many years of repeated cystoscopies and subsequent surgeries, imaging, and associated bother. Many biomarkers were proposed to supplant more invasive procedures and mitigate patients’ distress, but none of them have passed the test of time. Currently, three measures seem promising and remain under scientific scrutiny: ADX Bladder using mini chromosome maintenance 5 urine expression; Bladder EpiCheck testing DNA methylation; and Xpert Bladder Cancer Monitor, a targeted mRNA based (ABL1, CRH, IGF2, UPK1B, ANXA10) urine test. It has been shown that all three assays are able to rule out HG NMIBCA with certainty well above 90%. However, until proven otherwise, cystoscopy remains the mainstay procedure in the surveillance protocol for patients with NMIBCa. If diagnosed with low-grade disease previously, one may discriminate high-grade and low-grade lesions under the scope with decent accuracy [19]. In those with suspected low-grade cancer unwilling to undergo subsequent TUR within the regular operating room, office fulguration or even surveillance are advocated by some authors. The DaBlaCa-13 study group has proposed another alternative for these patients [20]. In this pivotal, randomized study, chemoablation with six intravesical instillations of mitomycin C, over 2 weeks for recurrent intermediate- and high-risk Ta NMIBC resulted in complete response in 57% of cases.

Bacillus Calmette-Guerin (BCG) immunotherapy is the only one conservative measure that has been proved and used for decades to prevent progression in high-risk NMIBCa. Although shown to be efficient, the optimal schedule, timing, dosing, strains, and the definition of failure remained under protracted dispute over the years. In EORTC-GU trial (EORTC 30962), the optimal dose of BCG has been tested [21]. When compared to the full dose, dose reduction by 2/3 is not followed by lower morbidity of instillations, and in those diagnosed with high-risk NMIBCa, one-year therapy is inferior to three-year therapy with respect to disease recurrence if the full-dose BCG was administered. However, the risk of BCa progression in these subgroups remained unchanged. Soon after the publication, the urological community faced the global BCG shortage, which resulted in restless search for any possibility to decrease the demand for the adjuvant treatment. Instead of a dose reduction, a limited number of BCG instillations was tested in NIMBUS trial [22]. In this multicenter, noninferiority study patients received nine doses, with three of them provided as an induction course (at weeks 1, 2, and 6) and the rest as the maintenance at months 3, 6, and 12. The control group received standard 15 BCG instillations. The trial has been stopped prematurely due to strikingly unfavorable results reflected by the shorter time to recurrence observed in the experimental arm. In summary, the evidence indicates that the instillation intensity is much more important than the dose of BCG in bladder cancer recurrence prevention. 

In 2018, FDA has summarized the evidence and proposed the definition of BCG unresponsive disease. It is to be diagnosed only if either five of six doses of an induction course followed by at least two of three doses of maintenance therapy or two of six doses of a second induction course [23]. This has set the stage for the future studies devoted to BCG failure, the phenomenon known for dismal prognosis. In the group of those with T1G3 BCa, 1- and 5-yr disease-progression rates mounted to 11.4% and 19.8%, respectively, and corresponding 1- and 5-yr disease-specific death rates halved the progression rates and equaled 4.8% and 11.3% [24].

**Figure 1:**
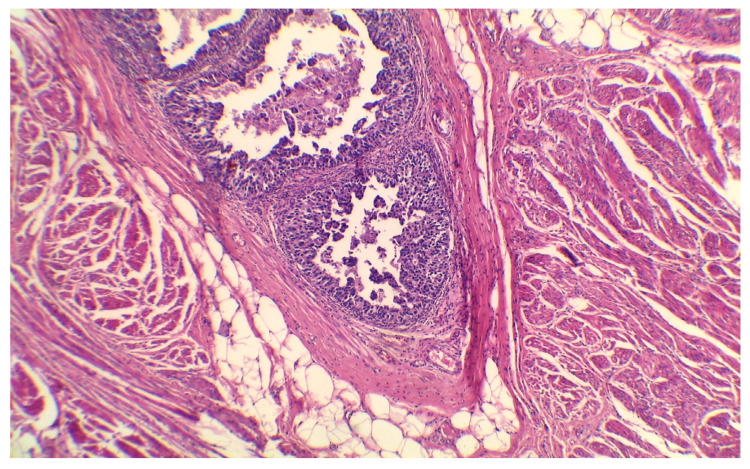
Lymph-vascular invasion of urothelial BCa. H&E stain, 50× magnification. Courtesy of M. Pyzlak, MD, PhD.

**Figure 2:**
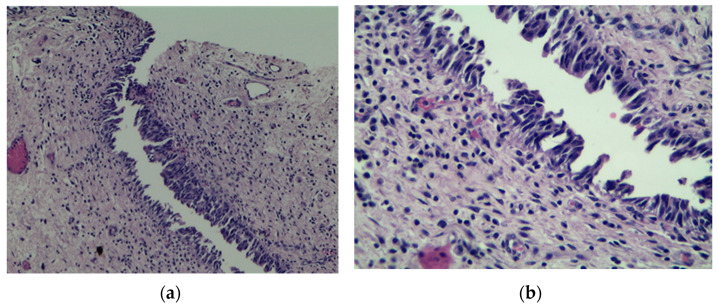
Micropapillary variant of urothelial bladder cancer, H&E stain. A phenomenon associated with dismal prognosis; (**a**) 100×; (**b**) 200×. Courtesy of M. Pyzlak, MD, PhD.

Several unfavorable predictive factors for disease progression in T1G3 BCa patients treated with BCG have been recognized so far. Besides well-established tumor-dependent factors such as concomitant CIS and tumor size, lymphovascular invasion (LVI) (Figure 1) and some forms of variant histology of urothelial carcinoma (VH) (Figure 2) are also linked with worse prognosis. It was estimated that the five-year risk of progression mounts to 38.7%, 47.3%, and 78.3% for patients with LVI, VH, and both features, respectively, compared to 7% for those with none of the above factors [25].

Moreover, an association between BCG refractoriness and metabolic disorders has been recently postulated [26]. Insulin resistance and low-grade systemic inflammation seem to be factors bridging metabolic syndrome to poor clinical outcome in T1G3 BCa. This could be explained on the basis of insulin, IGF-1, cytokines and growth factors’ effects on cell proliferation [27,28]. Several blood-derived biomarkers of systemic inflammation have been tested against usefulness in predicting BCa recurrence [29] and progression [30]. Combination of neutrophil/lymphocyte ratio, platelet/lymphocyte ratio and monocyte/lymphocyte ratio in group of patients with high-risk NMIBCa has prognostic value for both recurrence, progression, and cancer-specific survival [30]. Systemic inflammation and hyperinsulinemia hypothesis could therefore explain worse outcomes in overweighted and diabetic patients with NMIBCa. Based on the results of a retrospective study, overweight (HR: 2.52, *p* < 0.001) and obesity (HR: 2.521, *p* < 0.001) were significantly correlated with an increased risk of progression in BCG-treated T1G3 BCa patients [31]. Similarly, patients with type 2 diabetes mellitus have significantly higher risk of disease recurrence (HR: 1.41) and progression (HR: 1.27) [32].

Radical cystectomy is the mainstay therapy for patients who progressed despite the BCG treatment. For those who deny removal of the bladder or cannot be subjected to the surgery for number of reasons, there is an urgent need for a conservative alternative to intravesical immunotherapy that would salvage the patients who progress after BCG. So far, two novel agents have been shown to be successful and FDA approved in the management of BCG failure: pembrolizumab and nadofaragene. A number of trials are under way and new drugs will hopefully arrive soon (Table 1).

### 3.3. Muscle Invasive Bladder Cancer

If left without treatment, the majority of patients with MIBCa succumb to the disease within two years of diagnosis [33]. Therefore, radical removal of the bladder (RC) with concomitant meticulous pelvic lymph node dissection has become the gold standard way of management of muscle-invasive bladder cancer for many decades. Surprisingly, the value of lymphadenectomy has been recently challenged and the results of ongoing randomized trial are awaited [34]. As the majority of patients recur with distant metastases and half die of BCa within five years despite RC, perioperative chemotherapy that target micrometastases has gained widespread acceptance. Indeed, cis-platin based, combination neoadjuvant chemotherapy (NAC) has been proved to prolong overall survival of patients subjected to radical cystectomy due to muscle-invasive disease and has become routine care [35]. Despite the inherent delay, NAC brings a survival benefit to those with clinical T2, T3 or even T4 BCa. However, a third of patients subjected to NAC have non-responding disease confirmed by pathology of the surgical specimen [36]. Their prognosis is substantially worse than their responding counterparts. Therefore, markers that would predict BCa susceptibility to NAC or methods for interim response assessment to pursue with immediate surgery are urgently needed, as well as alternative agents in those not eligible for cis-platin-based therapy. 

Several authors have attempted to link the tumor molecular profile with its response to chemotherapy. Alterations in one or more of the three DNA repair genes (ATM, RB1, and FANCC) were associated with pathologic response and better overall survival [37]. Similar observations were found in relation to cell-cycle gene mutation status [38]. Combining 1750 transcriptomic profiles from 16 published datasets, a consensus molecular framework of MIBCa was introduced [39]. Six molecular classes of the disease were identified including luminal papillary, luminal non-specified, luminal unstable, stroma-rich, basal/squamous, and neuroendocrine-like. Consensus forms a robust classification that hopefully would be uniformly used in future studies in different clinical contexts. According to one such study, basal tumors were observed to provide the most significant improvement in OS with NAC compared to surgery alone, whereas luminal tumors were associated with the best outcome regardless of perioperative treatment [40]. However, in a randomized, phase II SWOG S1314 study, the score generated based on gene expression profile failed to predict the response to NAC in patients undergoing radical cystectomy [41]. This and other studies suggest that additional factors beyond genetic profiles must be taken under consideration to predict the response to NAC. Those who are not found with malignant tissue at final pathology (pT0) achieve excellent survival in contrast to those who have advanced disease. Regardless of the type of neoadjuvant regimen, consolidative surgery provides a unique and definitive source of histological material for a number of studies in this respect. TURBT performed amid subsequent courses of systemic treatment may further enhance our understanding of the cancer biology in particular cases. A lack of visible lesions at TURBT does not necessarily predict pT0 at RC. As much as 64% of all cT0 cases harbor residual disease and a quarter of them have advanced cancer [42]. However, if combined with modern imaging such as multiparametric magnetic resonance imaging (mpMRI), interim TUR may become an accurate assessment tool to confirm the response to ongoing neoadjuvant systemic therapy. The results of the PURE-01 study suggest that mpMRI with its VI-RADS score (vesical imaging reporting and data system) has the capability to discriminate pT0 stage in those subjected to immune therapy before RC [43]. 

The therapeutic landscape for patients with advanced disease is constantly expanding with immune-checkpoint inhibitors and pan FGFR inhibitor approved by FDA in recent years. Their success in metastatic disease forces many investigators to look into their potential role in earlier stages of bladder cancer (Table 2). An immune picture of MIBCa emerges from the trials and reveals the complexity of the relationship between the cancer and its host, making the disease more prone to a particular therapeutic strategy. At least seven aspects of this specific interplay were introduced and include tumor foreignness, immune cell infiltration, the absence of inhibitory checkpoints, general performance and immune status, absence of soluble inhibitors, absence of inhibitory tumor metabolism, and tumor sensitivity to immune effectors [44]. Potential candidates for the use of pembrolizumab or erdafitinib are recommended to be subjected to up-front evaluation of PD-L1 expression or FGFR2/3 profile, respectively. These have made our uro-oncological community enter the era of individualized therapy of bladder cancer.

## 4. Conclusions

Advances in the bladder cancer diagnosis and management have improved our understanding of the disease biology. Social awareness of risk factors together with decreased occupational exposure and a vigilant approach to the diagnosis of the disease will reduce the amount of newly diagnosed advanced cases. High-quality transurethral surgeries with more efficient and less morbid adjuvant therapies will reduce the caseload of radical cystectomies. Finally, the molecular picture of advanced disease revealed by sophisticated technology will tailor systemic therapy. To provide a favorable outcome, the treatment of bladder cancer will require constant mutual interplay among a variety of medical professions.

## Figures and Tables

**Table 1:** Ongoing trials on treatment failure after intravesical BCG immunotherapy.

Drug	Condition	Phase	Status	Estimated Study Completion Date	Study Number
Pembrolizumab + BCG	Reccurent high risk or BCG-refractory NMIBC	1	Active, not recruiting	February 2022	NCT02808143
Durvalumab	BCG-refractory NMIBC	2	Recruiting	31 December 2021	NCT03759496
INSTILADRIN (nadofaragene firadenovec)	BCG-unresponsive NMIBC	3	Active, not recruiting	31 August 2022	NCT02773849
Durvalumab	BCG-refractory CIS	2	Active, not recruiting	31 December 2021	NCT02901548
Atezolizumab	BCG-refractory NMIBC	2	Recruiting	31 December 2021	NCT02451423
Camrelizumab	BCG-refractory NMIBC	1, 2	Not yet recruiting	October 2022	NCT04706598
Erdafitinib	NMIBC recurrence after bacillus Calmette- Guerin (BCG)	2	Recruiting	20 June 2026	NCT04172675
Atezolizumab	BCG-unresponsive NMIBC	2	Active, not recruiting	1 August 2021	NCT02844816
Gemcitabine and Pembrolizumab	BCG-unresponsive NMIBC	2	Recruiting	31 March 2023	NCT04164082
Durvalumab monotherapy Durvalumab + BCG Durvalumab + EBRT	BCG-relapsing tumour	1, 2	Recruiting	1 March 2023	NCT03317158

**Table 2:** Ongoing trials on perioperative (radical cystectomy) systemic treatment.

Drug	Type of Intervention	Condition	Phase	Status	Estimated Study Completion Date	Study Number
Atezolizumab	Neoadjuvant	MIBC in subjects appropriate for cystectomy and refusing or ineligible for neoadjuvant chemotherapy	2	Recruiting	31 December 2021	NCT02451423
Bintrafusp alfa	Neoadjuvant	cT2-T4aN0-1M0	2	Not yet recruiting	January 2025	NCT04878250
Gemcitabine, Avelumab and Carboplatin	Neoadjuvant	cT2-T4aN0M0	2	Not yet recruiting	30 April 2029	NCT04871529
Tislelizumab and Nab-Paclitaxel	Neoadjuvant	cT2-4aN1-3M0	2	Recruiting	July 2024	NCT04730219
Retifanlimab or epacadostat or combination	Neoadjuvant	cT2-T3b, N0, M0	2	Not yet recruiting	30 June 2024	NCT04586244
Olaparib and Durvalumab or Durvalumab monotherapy	Neoadjuvant	cT2-T4aN0M0 or cT1-T4aN1M0, ineligibility for cisplatin chemotherapy	2	Not yet recruiting	December 2026	NCT04579133
Tislelizumab monotherapy or Tislelizumab + Gemcitabine and Cisplatin	Neoadjuvant	cT2-T4NxM1 and oligometastatic disease (solitary organ metastasis and ≤3 metastatic lesions and ≤5 cm metastasis diameter and no liver metastasis)	2	Recruiting	1 December 2024	NCT04570410
Toripalimab and Gemcitabine	Neoadjuvant	Clinical stage II-IIIB, ineligibility for cisplatin chemotherapy	2	Recruiting	December 2023	NCT04553939
Gemcitabine and Cisplatin + Toripalimab	Neoadjuvant	cT2-4aN0M0	2	Recruiting	1 October 2022	NCT04099589
Dose dense MVAC with pegylated GCSF	Neoadjuvant	cT3-4a and N1-3	2	Recruiting	31 December 2021	NCT04047693
Durvalumab + GC or Durvalumab + Carboplatin Gemcitabine or Durvalumab + DD-MVAC	Neoadjuvant	variant urothelial carcinoma histologies cT2-T4aN0-N1M0	2	Recruiting	13 August 2022	NCT03912818
Abemaciclib	Neoadjuvant	cT2-T4, pure or mixed histology urothelial carcinoma, ineligibility for cisplatin chemotherapy	1	Recruiting	February 2024	NCT03837821
Atezolimumab	Neoadjuvant	MIBC	-	Not yet recruiting	31 May 2022	NCT03577132
Durvalumab + ddMVAC or Durvalumab + Tremelimumab + ddMVAC	Neoadjuvant	MIBC	1, 2	Recruiting	September 2025	NCT03549715
Nivolumab + EBRT	Neoadjuvant	cT3/4 cN0/N+ M0	2	Recruiting	August 2022	NCT03529890
Ipilimumab + Nivolumab	Neoadjuvant	cT3-4aN0M0 OR T1-4aN1-3M0	1	Active, not recruiting	1 September 2021	NCT03387761
Pembrolizumab	Adjuvant	pT2 and/or N^+^	3	Recruiting	1 June 2025	NCT03244384
Atezolizumab + GC	Neoadjuvant	cT2-4a;N0/X;M0	1, 2	Active, not recruiting	December 2021	NCT02989584
Nivolumab or Nivolumab + Urelumab	Neoadjuvant	T2-T4N0-N2M0, subjects ineligible for or refusing cisplatin chemotherapy	2	Recruiting	March 2022	NCT02845323
Pembrolizumab	Neoadjuvant	T2-T4a N0 M0	2	Recruiting	27 December 2021	NCT02736266
Pembrolizumab + GC	Neoadjuvant	cT2-4aN0M0, ineligibility or eligibility for cisplatin chemotherapy	1, 2	Active, not recruiting	July 2024	NCT02365766

ddMVAC—dose-dense methotrexate vinblastine doxorubicin cisplatin; GC—gemcitabine cisplatin.

## Data Availability

All data are included in the main text.
